# Ultrahigh Temperature Purification of Graphite for the Development of a Continuous Process

**DOI:** 10.1021/acsomega.5c05566

**Published:** 2025-09-16

**Authors:** Yewen Tan, Marc Duchesne, Anna Doninger, Matthew Meyers, Igor V. Barsukov

**Affiliations:** 1 113679 Natural Resources Canada, CanmetENERGY in Ottawa, 1 Haanel Drive, Ottawa, Ontario K1A 1M1, Canada; 2 American Energy Technologies Co., 265 Alice Street, Wheeling, Illinois 60090, United States

## Abstract

This work presents a study of ultrahigh temperature purification of natural Canadian graphite flakes. The concentrated natural graphite flakes were purified using two test facilities, an ultrahigh temperature fixed bed furnace and an ultrahigh temperature fast-heating counterflow reactor. With the fixed bed furnace, the natural graphite flakes were purified at 2500 or 2800 °C for 15–120 min. With the counterflow reactor, the residence time was ∼20–25 min, with an average temperature of 2700 °C and higher local temperatures due to electric arcing. The heat-treated samples were characterized by using several different analysis techniques. The results showed that the samples treated with the fast-heating counterflow reactor reached a very high purity above 99.9 wt % carbon. The samples treated at 2800 °C in the fixed bed furnace reached a similar purity. At the lower temperature of 2500 °C, a similar purity could only be achieved with a duration of at least 60 min. Four elemental analysis techniques to quantify impurities in graphite were evaluated in this work, with a focus on elements that disrupt the performance of Li-ion batteries, such as magnesium, aluminum, iron, copper, and silicon. The analysis results with the original graphite flakes and the heat-treated graphite flakes showed that significant differences exist among the various analysis techniques. For some critical elements, such as iron and silicon, the detected concentrations could differ by more than 1 order of magnitude.

## Graphical abstract

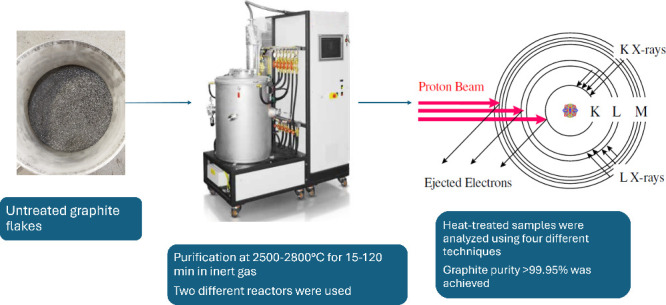

## 1 Introduction

High purity graphite is an important material and has many applications for a variety of industries. It is an important material in the nuclear industry. Also, it is used as the active material in negative electrodes of electrochemical power systems, such as lithium-ion batteries, as well as in cathode conductivity diluents used in traditional battery chemistries, including lithium primary, metal–air, and alkaline zinc–manganese dioxide. Production of solar cells and wafers for use in semiconductor devices requires ultrahigh purity graphite. Graphite is also employed as an electrode material for electric arc furnaces, electric discharge machining, and electrolysis.^1^

When natural graphite is mined, it usually has a low graphitic carbon content. Mined natural graphite undergoes several stages of mechanical separation, including flotation, to produce concentrated graphite with up to ∼95% total graphitic carbon.^2–4^ For many applications, this concentrated graphite must be purified to above 99.95 wt % carbon. There are several methods used to purify graphite. The advantages and disadvantages of the common methods have been reviewed and are summarized in Table 1.^3,4^

**Table 1:** Summary of the Common Graphite Purification Methods

purification methods	advantages	disadvantages
Alkali acid	Easy operation and relatively low production cost.	Vast consumption of process water, equipment corrosion issues, and generation of toxic wastewater.^5^
Hydrofluoric acid	Can produce high purity graphite.^3^	Toxic to human health and the environment.^6^
Carbochlorination, ∼1500 °C	Can produce high purity graphite.^7^ Good product yield.	Chlorine gas is costly, toxic, and corrosive.
Ultrahigh temperature (Acheson and Castner furnaces), ∼3000 °C	Can produce high purity graphite.^8^ No harsh chemicals needed.	High equipment costs and electricity consumption. Long production cycle.

Ultrahigh temperature purification can produce high purity products with relatively low environmental burdens when using clean electricity; as this method does not use corrosive chemicals, it does not produce associated toxic waste products. In addition, ultrahigh temperature processes can increase the crystallinity of carbon.^9^ Hence, beyond purifying natural mined graphite, they can alternatively produce synthetic graphite.^10–12^

The disadvantages of the conventional thermal purification methods are the high equipment costs, long production cycle, and high electricity consumption to maintain operating temperatures as high as 3000 °C. Hupp et al. estimated that, depending on the purity of the graphite required, the energy consumption in an Acheson furnace was ∼4.5 kWh/kg to produce graphite electrodes commonly used in open-arc furnaces. The energy consumption increases to 9 kWh/kg for nuclear grade graphite.^13^ According to Shen et al., the integrated energy consumption by some commercial Acheson units in China was as high as 15–18 kWh/kg of finished battery-ready synthetic graphite.^1^ Other sources indicated that the graphitization process using an Acheson furnace in China requires energy consumption of over 12 kWh/kg.^14^ The ultrahigh temperature Acheson process is also time-consuming and cannot be used for continuous operation; the entire heating and cooling cycle can take several weeks.^1,13^ Castner furnaces, due to their direct heating mechanism, have improved power efficiency with an energy consumption of less than 4.4 kWh/kg of graphite, and the production cycle can be reduced to less than a week.^13^

Fluidized beds enhance heat transfer, have a high heating rate at greatly enhanced processing efficiencies, and can be used for continuous operation so they can achieve a higher energy efficiency compared to Acheson and Castner furnaces. Fedorov et al. explored the feasibility of purifying graphite in an electrically heated fluidized bed using the direct Joule heating principle.^15–17^ Remaining knowledge gaps hinder the broad implementation of this technology for the purification of natural graphite flakes. These include an understanding of graphite flake fluidization,^18^ and the effects of time and temperature on purification. Although multiple studies covered purification temperatures from 1500 to 3000 °C and treatment durations from 10 min to 24 h,^1,8,9,12,19,20^ few studies have considered ultrahigh temperature purification of natural graphite flakes using residence times and heating rates that are representative of commercially practiced fluidized bed processing.

In this work, natural graphite flakes were purified using two different lab-scale, ultrahigh temperature systems to address the primary objective of this work, which is to evaluate the effects of the treatment temperature, duration, and heating rate on the reduction of impurity levels in graphite for conditions pertinent to fluidized bed purification. This is believed to be the first published study on the impact of heating rate and heating duration on the purification of Canadian natural flake graphite flakes above 2400 °C. Thermodynamic equilibrium calculations were performed to elucidate the removal of several elements by high temperature treatment in argon and nitrogen atmospheres.

The secondary objective of this work is to compare various graphite analysis methods from the point of view of quantifying parts per million-level concentrations of mineral impurities in refined graphite. The loss on ignition (LOI) method is typically used to determine the total concentration of all impurities in graphite. If graphite is to be used for advanced lithium-ion batteries, it must meet high quality specifications, not only on the overall carbon content but also on the permitted amounts of specific impurities. The most frequently used methods to quantify concentrations of individual elemental impurities are energy dispersive X-ray fluorescence spectroscopy (ED-XRF), glow discharge mass spectrometry (GDMS), inductively coupled plasma optical emission spectroscopy (ICP-OES, sometimes referred to as inductively coupled plasma-atomic emission spectroscopy), electrothermal vaporization with ICP-OES (ETV-ICP-OES), and inductively coupled plasma with mass spectrometry (ICP-MS). Proton-induced X-ray emission spectroscopy (PIXE) is emerging as a viable cost-effective alternative. Most of the published work involving graphite purity uses one of the analysis methods listed above. Mayer compared ED-XRF, ICP-OES and GDMS for purified graphite analysis.^21^ They concluded that, for purified graphite, ED-XRF was not suitable due to its inadequate detection limit, while ICP-OES and GDMS both have the required detection limits for purified graphite and gave similar results. Conversely, ASTM International has a standard method based on ETV-ICP-OES (ASTM D8186-18) for graphite impurities analysis.^22^ In the current study, we compare results from ICP-OES, GDMS, ETV-ICP-OES and PIXE. This is believed to be the first published study comparing all of these methods directly for graphite. Graphite characterization is supplemented with LOI analysis for the carbon content and X-ray diffraction (XRD) for the crystal structure.

## 2 Materials and Methods

### 2.1 Materials

The natural graphite flakes used in this study were provided by Nouveau Monde Graphite in Quebec, Canada. The flakes were concentrated using flotation to a total graphitic carbon (TGC) content of about 95%. The graphite came in different size distributions. For this work, Jumbo (∼300–600 μm), Large (180–425 μm), and Medium (∼75–180 μm) size fractions of graphite flakes were used. Size ranges reported here are from sieving following ASTM E11. Detailed sizing of the graphite flakes can be found in another study.^18^

### 2.2 Graphite Analysis Methods

Untreated and purified graphite samples were analyzed using five methods: loss on ignition (LOI) by SGS Canada using the ASTM C561-23 method, GDMS by Eurofins EAG Laboratories, ETV-ICP-OES by American Energy Technology Corp. (AETC), ICP-OES by SGS Canada, and PIXE by Elemental Analysis Inc.

Modulated fast-flow GDMS was used with indium as a binder. The detection limit for the elements of interest in this work was below 0.05 ppm. GDMS has the advantage of limited sample preparation requirements, as measurements are often carried out directly on the initial sample material.

In the ETV-ICP-OES method, the ICP and ETV were first calibrated together by loading 25 μL of two standards. After calibration, graphite flakes (∼20–40 μg) were loaded into a pretreated graphite crucible; pretreatment is aimed at removing any residue which might have deposited on the crucible or was left from a previous run through the instrument. The crucible is inserted into a resistively heated graphite furnace and brought to a temperature of 3000 °C while nonreclaimed R12 Freon is dispersed over the graphite flakes. Fluorine from the R12 Freon reacts with impurities, causing them to sublimate and get carried into the ICP unit. The detection limit with AETC’s ETV-ICP-OES is on the order of 0.01 ppm.

For the ICP-OES method used by SGS Canada, trace impurities were determined following lithium metaborate fusion of the residue obtained by ashing (ASTM C561-23). With this method, the detection limits for various elements are listed in Table 2.

**Table 2:** ICP-OES Elements Detection Limits

element	detection limit, ppm	element	detection limit, ppm
Al	10	Mo	4
Ba	0.3	Na	5
Ca	10	Ni	4
Co	2	P	20
Cr	4	Si	30
Cu	10	Ti	0.8
Fe	20	V	1
K	8	Zn	20
Mg	3	Zr	0.3
Mn	0.4		

For PIXE, the sample is ground to below 200 mesh and pressed into a pellet before being inserted into a vacuum/helium chamber equipped with a General Ionex 4 MV tandem accelerator with a duoplasmatron source capable of producing beam currents in the range of a few nanoamps to tens of microamps, a dual quadrupole focusing lens, an *x*–*y* beam scanner to ensure beam homogeneity, and a beam pulser with 50 ns response time. The detection limit for an element depends upon its concentration.^23^

XRD was used to characterize the graphite structure. Diffraction patterns were collected using a Rigaku Ultima IV XRD automated spectrometer over the angular range of 3–90° 2θ, with a step size of 0.02° and a scanning rate of 1° min^–1^. The system operates in the θ/θ geometry, utilizing CuKα radiation (λ = 1.540 598 1 Å), and is equipped with a diffracted-beam monochromator and a high-speed semiconductor one-dimensional X-ray detector (D/teX Ultra). Diffraction peaks of crystalline phases were identified by using the International Center for Diffraction Data (ICDD) database and Jade Plus version 7.5 software.

### 2.3 Ultrahigh Temperature NRC Reactor Purification

A high temperature, externally heated, fixed bed furnace (Carbolite LHT GR) was operated by the National Research Council of Canada (NRC). It will be termed the NRC reactor. Natural graphite flakes, in an amount of ∼100 g, were loaded into a graphite crucible. A lid with an opening was placed in the crucible. The crucible was then placed in the high temperature furnace with a maximum operating temperature of 3000 °C. Once the sample was loaded and the furnace closed, it was vacuumed and then flushed with argon. The vacuum and argon flush steps were repeated to ensure all of the air was removed from the furnace. The furnace was then filled with argon, and heating started at a rate of 16 °C/min with argon as a carrier gas with a flow rate of ∼2.3 NL/min. Once the target temperature was reached, the sample was held at that temperature for a predetermined duration. The sample was then cooled to room temperature in argon at a rate of 20 °C/min. Table 3 summarizes the test conditions.

**Table 3:** Test Conditions with the NRC Reactor

Jumbo flakes		Medium flakes	
temperature, °C	duration, min	temperature, °C	duration, min
2500	30, 60, 120	2500	30, 60, 120
2800	15, 30, 60	2800	15, 30

In this paper, the tests will be identified in such a way that 2500J30 refers to the Jumbo flakes treated at 2500 °C for 30 min, and the Medium flakes will be identified with M instead of J.

Literature data have shown that graphite purification can start at temperatures as low as 1500 °C,^20^ so when a sample is subjected to heat treatment in the furnace, its actual purification duration should thus account for the time during temperature ramping (up and down). When calculated in this way, the untreated graphite samples were exposed to temperatures above 1500 °C for an additional 112.5 min when the target hold temperature was 2500 °C, and an additional 146.25 min when the target hold temperature was 2800 °C.

### 2.4 AETC Ultrahigh Temperature Arcing Reactor

The other purification system used in this work is a bench-scale arcing reactor operated by AETC, which will henceforth be termed the AETC reactor. This reactor is an ultrahigh temperature continuous fluidized bed reactor that takes advantage of electrical arcing for heating. The inside of the furnace is composed of several sections, which include a cooling jacket that surrounds the entire reactor cylinder with recirculated cooling water/glycol, a layer of thermal insulation, graphite bricks, and a cylindrical graphite electrode. These individual sections are represented in Figure 1. The empty section between the electrode and the graphite brick layer is where the material is heated in a fluidizing nitrogen atmosphere.

**Figure 1:**
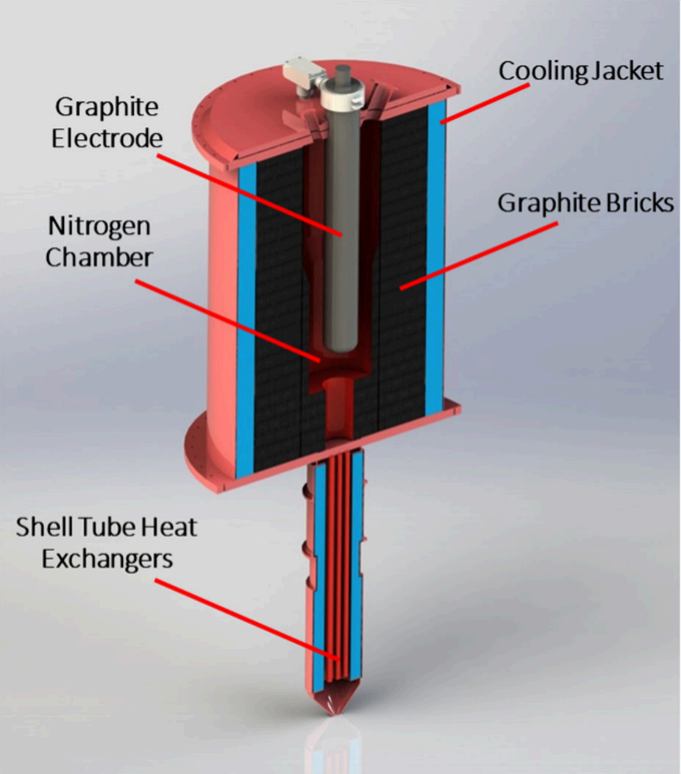
Cross-sectional model of AETC’s ultrahigh temperature purification furnace.

In the process of going through the reactor, natural graphite has a residence time of ∼20–25 min and reaches an average temperature of ∼2700 °C, with higher temperatures near the electrical arcs. The reactor can process up to 10 kg/h of graphite and is outfitted with a boron-silicate glass, allowing the observation of the inside of the reactor. During operation, innumerable electrical arcs inside the reactor can be observed. Heat-treated graphite goes through a series of heat exchangers, where it is cooled and conveyed to subsequent stages of material processing. Mineral impurities in the gas form are picked up by the ascending stream of nitrogen and carried away from the reactor to an afterburner and then into a scrubber.

## 3 Results

### 3.1 Characterization of Graphite Flakes Prior to Purification

Figure 2 shows the ash content of the untreated graphite samples. It is observed that the ash content increased as flake size decreased, which is commonly observed;^24^ this is likely due to the higher surface area to volume ratio of smaller flakes. Impurities in graphite ore are minerals that are finely disseminated within the rock matrix. During natural formation and subsequent mining, these impurities can become attached to or embedded within the graphite flakes. Smaller graphite flakes, due to their larger surface area relative to their volume, have a higher probability of having these mineral impurities embedded within. As well, impurities tend to reside on the surface or along the edges and defects of the graphite flakes, so a relatively larger proportion of a small flake’s mass is exposed to potential contamination compared to a large flake.

**Figure 2:**
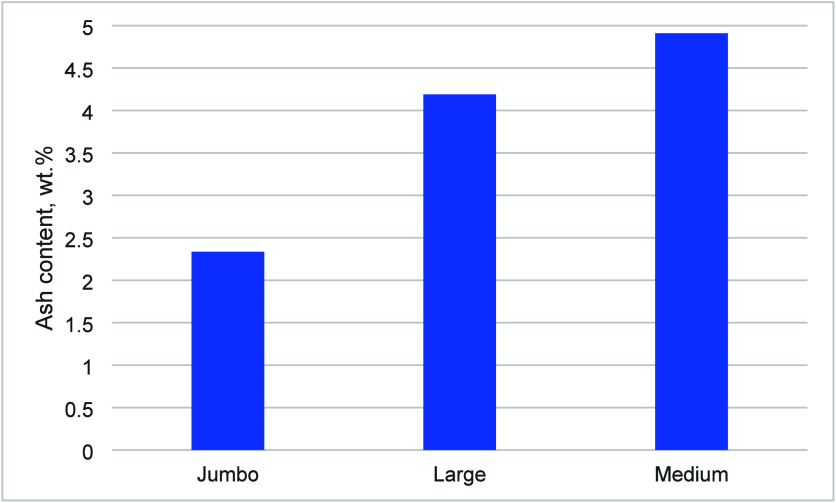
Ash content of untreated graphite samples, in wt %.

Figure 3 shows the impurities in the untreated Medium and Jumbo flakes. The full set of impurities analysis results with the four analytical methods is shown in Table S1 in the Supporting Information. For ICP-OES, duplicate analyses were done for Medium and Large flakes, and triplicate analyses were done for the Jumbo flakes. For PIXE, duplicate analyses were done for all three flake types.

**Figure 3:**
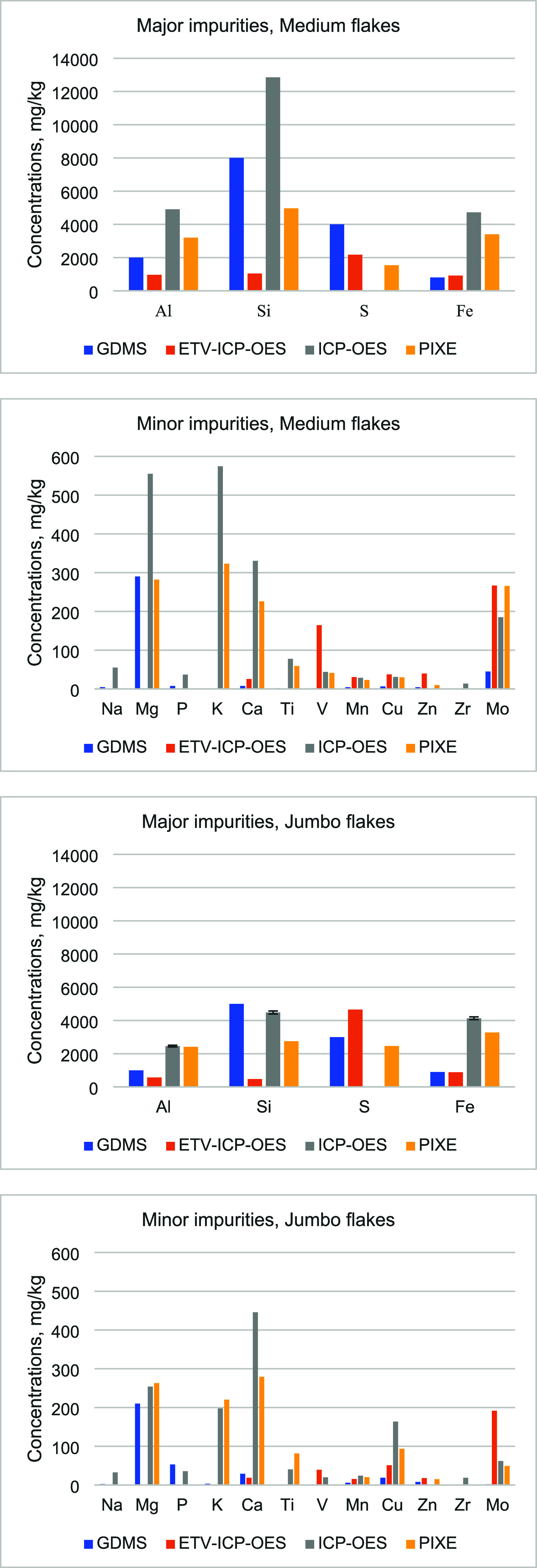
Impurities in the untreated Medium and Jumbo graphite flakes determined using four analytical methods.

One notable element missing from the ICP-OES results is sulfur due to sulfur loss during sample preparation for ICP-OES. While unconventional sample preparation methods can be used to avoid this loss, these were not applied in the current study.

Sodium was not reported with PIXE. The concentration of Na has a very high X-ray response cross-section, but because the energy of its X-ray is so low, it is severely absorbed and, therefore, has the highest detection limit for PIXE analysis.

Overall, Table S1 shows that the graphite is characterized by high amounts of S (as confirmed by GDMS, ETV-ICP-OES, PIXE), Si, and Al (as detected by GDMS, ICP-OES, PIXE), as well as Fe (as measured by ICP-OES, PIXE). Among minor impurities, the presence of Mg (GDMS, ICP-OES, PIXE), Mo (ETV-ICP-OES, ICP-OES, PIXE), Ti (ICP-OES, PIXE), Na (ICP-OES), Ca (ICP-OES, PIXE), K (ICP-OES, PIXE), and V (ETV-ICP-OES) were observed.

### 3.2 Purification of Natural Graphite with the NRC Reactor

Thermally purified samples retrieved from the NRC reactor were analyzed for the ash content. The results are shown in Table 4, which additionally shows the duration of time that the sample was exposed to temperatures above 1500 °C.

**Table 4:** Ash Content (in wt %) of the Samples Purified in the NRC Reactor

	time above 1500 °C, min	SGS Canada (ASTM C561-23), wt %
2800J15	161.25	<0.01
2800J30	176.25	<0.01
2800J60	206.25	<0.01
2500J30	142.50	0.15
2500J60	172.50	0.01
2500J120	232.50	<0.01
2800M15	161.25	<0.01
2800M30	176.25	<0.01
2500M30	142.50	1.25
2500M60	172.50	0.86
2500M120	232.50	0.26

Table 4 shows that samples purified at 2800 °C reached very high purity. At 2800 °C, 15 min of hold time is sufficient to reach ultrahigh purity (>99.99 wt % carbon). Jumbo flakes were purified to ultrahigh purity (99.99 wt % carbon) at 2500 °C for 60 and 120 min. For Medium flakes, the purity obtained at 2500 °C is noticeably lower, even at 120 min of exposure time (99.74 wt % carbon).

The purified samples were analyzed for residual impurity elements using GDMS, ICP-OES, ETV-ICP-OES, and PIXE. The ICP-OES results are not presented because all impurity levels are below its detection limits. The complete analysis results for the heat-treated graphite flakes are shown in Figure 4 and in Tables S2 and S3 in Supporting Information.

**Figure 4:**
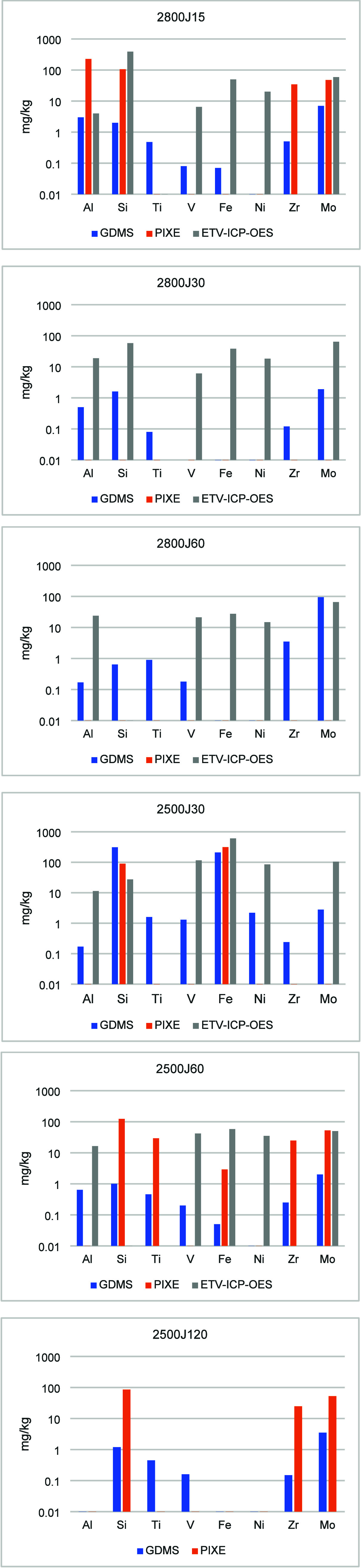
Analysis of Jumbo flakes purified in the NRC reactor (concentration of impurities are listed in mg/kg).

Tables S2 and S3 and Figure 4 show that all three methods show the same trend, in that higher temperature and longer duration lead to lower impurity concentrations in the samples. However, each individual impurity’s concentration can vary considerably with different analysis methods.

Overall, the results from the NRC reactor tests confirm that heat treatment is an effective way to purify the natural graphite samples used in this work for disruptive elements such as Mg, Al, Fe, Cu, and Si, especially at 2800 °C.

### 3.3 Purification of Natural Graphite with the AETC Reactor

The ash contents for Medium, Large, and Jumbo flakes purified using the AETC reactor are all below 0.01 wt %. The graphite flakes purified with the AETC reactor were analyzed using GDMS, ETV-ICP-OES, PIXE, and ICP-OES (Figure 5 and Table S4 in Supporting Information). Zn, Pb, Cd, Co, and Cu were analyzed by all methods but not detected. The ICP-OES results are not shown because all elements are below the detection limits.

**Figure 5:**
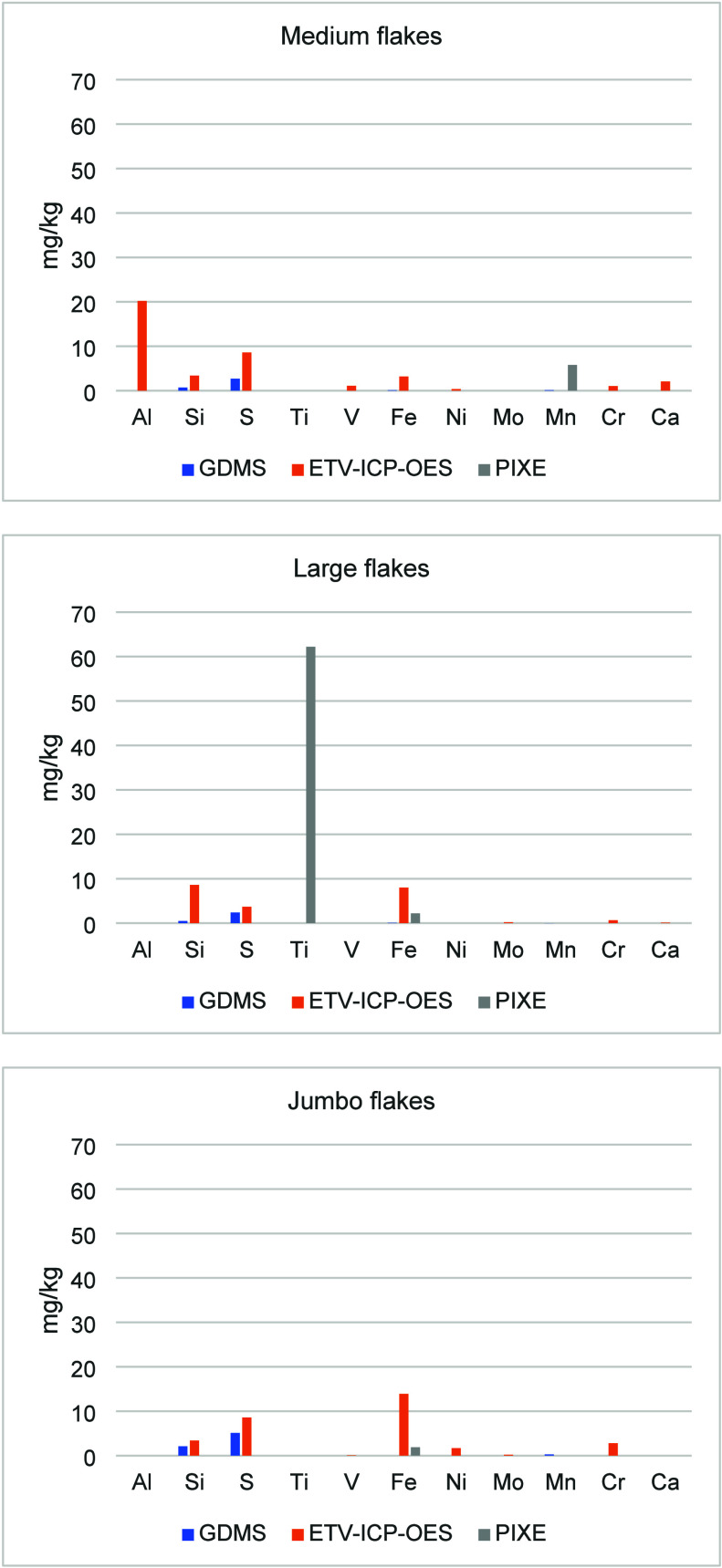
Analysis results for the samples purified in the AETC reactor (concentration of impurities is listed in mg/kg).

Figure 5 shows that all elements (including Fe, Si, and Mo, which have shown to be resistant to heat treatment in the NRC reactor) are reduced to very low concentrations, indicating that the AETC reactor conditions are more effective than the NRC reactor conditions in purifying the graphite.

XRD analysis, presented in Figures S1–S4 in the Supporting Information, showed that the interlayer distance of the Jumbo graphite samples varied from 0.335 to 0.337 nm, including the non-heat-treated natural graphite. This indicates that these samples have high crystallinity, considering that the interlayer distance of pristine graphite is 0.3354 nm.^25–27^

## 4 Discussion

### 4.1 Purification Conditions

The heating mechanisms for the NRC reactor and the AETC reactor are very different. In the NRC reactor, the untreated graphite is heated statically through the sample holder walls very gradually. Even as the furnace reaches its target temperature, it may still take some time for the graphite flakes to reach the set temperature. The more gradual heating in the NRC reactor might allow the formation of carbides that are more resistant to high temperature treatment.

With the AETC reactor, the graphite flakes are instantly heated to very high temperatures and stay at very high temperatures for the entirety of the residence time. For this reactor, even though the average bed temperature was ∼2700 °C, the arcing temperature could be much higher and greatly surpasses the boiling and sublimation points of impurities. The fast heating in the AETC reactor may inhibit the formation of carbides, unlike that in the NRC reactor. Therefore, while the graphite flake residence time in this reactor is shorter than in the NRC reactor, they can still be purified to a higher degree.

According to Wissler, many electrochemical applications require graphite purity with <0.1% ash and heavy metal elements concentration below 10 ppm, while some applications may require heavy metal elements concentration below 1 ppm.^28^ Table 4 shows that, according to ASTM C561-23, all the Jumbo flakes treated in the NRC reactor reached ash content below 0.01%, except 2500J30. For Medium flakes treated in the NRC reactor, a temperature of 2500 °C is insufficient to reach such a low ash content even at the longest holding time of 120 min. Results summarized in Table 4 show that the maximum operating temperature is a critical parameter for high temperature graphite purification.

For graphite used as an anode material in Li-ion batteries, the presence of certain impurities can be detrimental to performance. According to Fink et al., who studied the influence of four metallic contaminants (Fe^0^, Al^0^, Cu^0^ and Mg^0^) on the performance of Li-ion battery electrodes, Cu^0^ and Mg^0^ are the most disruptive anode contaminants.^29^ Fink et al. concluded that metallic impurities in anodes could react with Li, leading to a resistive solid electrolyte interface and consuming excess Li. These anode impurities also serve as weak catalysts to accelerate electrolyte decomposition. In our tests, Mg and Cu are completely removed with ultrahigh temperature treatment.

Ni Yingping et al. pointed out that the presence of Fe was an important indicator of the efficiency of graphite anode materials. The charge transfer from the anode becomes more efficient as the Fe content becomes lower.^30^ According to Zhang et al., even though trace impurities like Cu and Fe have little effect on the initial discharge specific capacity of the graphite anode, they significantly reduce its cycling stability.^31^ These metals can undergo unwanted side reactions during battery operation, potentially leading to capacity degradation over time. Removing these impurities is crucial for improving the long-term performance. At least one graphite anode manufacturer specifies an Fe content of less than 30 ppm for high power applications.^32^

Nonmetallic contaminants can also impact Li-ion battery performance. The presence of Si is known to cause volume expansion, which can lead to a number of detrimental effects.^33^

It is noteworthy that graphite manufacturers often highlight low concentrations of Fe and Si as an indicator of high graphite quality.^34,35^

Our results with the NRC reactor show that Fe and Si are much more resistant to heat treatment compared to Mg and Cu. According to GDMS and PIXE, it is necessary to heat the sample to 2800 °C to reduce the Fe concentration to below 30 ppm if one wants to keep the treatment duration under 30 min. At 2500 °C, it is necessary to extend the treatment duration beyond 60 min to reduce the Fe content to below 30 ppm. ETV-ICP-OES gave slightly higher concentrations of Fe, although the trend is the same as with GDMS and PIXE.

With Si, there are more significant differences among the analysis methods. For example, for sample 2800J15, Si concentration measured by PIXE and ETV-ICP-OES is 50 and 200 times higher, respectively, than that measured by GDMS. Similar, yet less significant, discrepancies exist for other samples. It is unclear why there are such big differences for Si concentrations determined by different analysis methods. This observation reinforces the need to find a common standard method to measure impurities in highly purified graphite.

Results from ICP-OES, ETV-ICP-OES, and PIXE show that Mo is persistent in the NRC purified samples. As well, the amount of Mo in the purified samples is sometimes higher than that in the untreated graphite. For example, according to ICP-OES, Mo in 2800M60 is 318 ppm, while it is ∼185 ppm in the untreated Medium flake. Another example is that of the GDMS result, which shows that the untreated Jumbo flakes contained 2.1 ppm of Mo while the purified sample (2800J60) contained 94 ppm of Mo. There is currently scant knowledge on the impact of trace amounts of Mo on Li-ion battery performance, whether positive or negative.

Overall, the test results conclusively show that graphite flakes treated in the AETC reactor reached higher purity than those treated in the NRC reactor. The results from the AETC reactor are especially encouraging for the development of a continuous process, because the heating of the graphite flakes is much more similar to an actual fluidized bed in terms of heating rate and the residence time in the ultrahigh temperature zone compared to the NRC reactor. A faster heating rate and effective agitation with a shorter residence time can greatly improve the efficiency of the graphite purification process, by avoiding the lengthy time needed for heating and cooling in the conventional high-temperature processes.

### 4.2 Thermodynamic Equilibrium Calculations

Thermodynamic equilibrium calculations were conducted using the Equilib module of FactSage to gain insights into how selected impurities evolve with temperature. These elements were selected either because they have a high concentration in the untreated graphite, such as Si, Al and Fe, or because they have shown to be difficult to remove with high temperature treatment, such as Ti, Mo, and Zr. Because NRC tests used argon gas and AETC tests used nitrogen gas, FactSage calculations were performed using both gases. The Equilib module of FactSage is based on thermodynamic equilibrium, so it does not provide direct information on the kinetics of impurities removal. Another limitation in the calculations is that potential interactions of various impurities were not considered. There is little information in the literature on the interactions of impurities.^1^ Despite these limitations, thermodynamic equilibrium calculations can provide valuable information, especially in identifying how readily each element can be removed by heating.

The FactSage curves are shown in Figure 6 for argon. Here, “[0.25–1.00]­X_[0.1 or 3]­Ar” should be read as below:

- “0.25–1.00” indicates the molar ratio of the impurity element to carbon (1.00 is without carbon, 0.75 is 75 mol % impurity with 25 mol % C, 0.25 is 25 mol % impurity with 75 mol % C)
- “X” indicates the impurity element (Ti, Mo, Si, Fe, Al, V, Ni, or Zr)
- “0.1 or 3” indicates the molar ratio of gas to carbon (0.1 or 3). The low and high molar ratios of gas were determined based on typical conditions during NRC testing (0.1 is ∼138 NL/h for 15 min, 3 is ∼138 NL/h for 266 min)
- “Ar” or “N2” indicates the atmosphere of argon or nitrogen

For example, 0.75Si_0.1Ar indicates a condition of 75% Si with 25% carbon exposed to an argon atmosphere for 15 min, whereas 0.25Si_3.0Ar indicates a condition of 25% Si with 75% carbon exposed to an argon atmosphere for 266 min.

**Figure 6:**
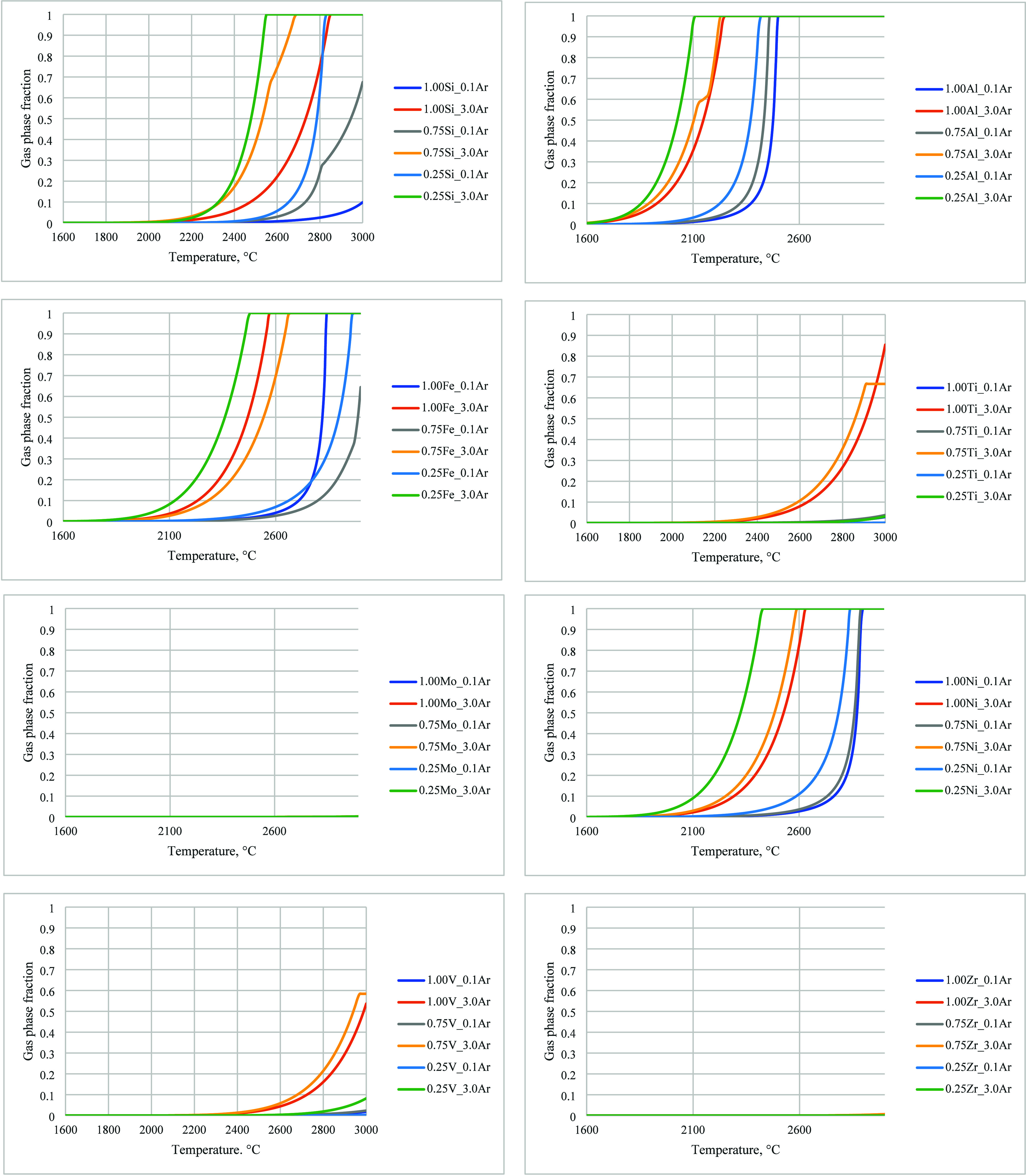
Evolution of the gas phase molar fraction of impurities with temperature in argon, with and without carbon, calculated by using FactSage.

These figures show that higher temperatures and higher gas molar ratios lead to higher levels of impurity removal. Adding carbon typically increases the fraction of impurities going to the gas phase, except for Fe and Ti. Because carbon is the dominant element in any graphite, curves with 25% impurity elements and 75% carbon are more relevant. The calculations showed that none of the impurities had over 10% removal at 1800 °C or cooler, and none of the impurities had over 50% removal at 2000 °C or cooler. Al is the easiest to remove, while Mo and Zr are the most resistant to high temperature removal. Ti and V are also resistant to high temperature removal, though both do turn into gas at a higher gas ratio.

In their work, Bao et al. discussed the formation of TiC and VC, both of which have a high decomposition temperature.^8^ Nevertheless, our experimental results did show that both Ti and V were removed to a high degree in the AETC reactor, though not completely. On the other hand, experimentally, Mo and Zr were almost not removed at all, which agrees with FactSage calculations.

According to the calculations, Fe and Si cannot be completely removed, even at 3000 °C, especially at a low gas-to-carbon ratio. However, our test results showed that these two elements were, in fact, effectively removed at 2800 °C and 15 min.

Our FactSage results differ from Lähde et al.’s calculations.^9^ Their calculations showed that most of the impurities could be removed below 2400 °C, while ours show that temperatures higher than 2500 °C are required for significant impurity removal, especially if the duration is short (cases with an argon-to-carbon ratio of 0.1). This agrees with our test results. One of the differences in Lähde et al.’s approach is that they used an argon-to-carbon molar ratio of 5:1 in their calculations, which is higher than ours. As our results have shown, increasing the argon-to-carbon molar ratio is generally favorable to impurities removal.

FactSage calculations show that changing the atmosphere from argon to nitrogen did not have significant effects on these curves except for Ti, whose gas phase fraction of Ti is predicted to be noticeably lower in nitrogen than in argon, as shown in Figure 7.

**Figure 7:**
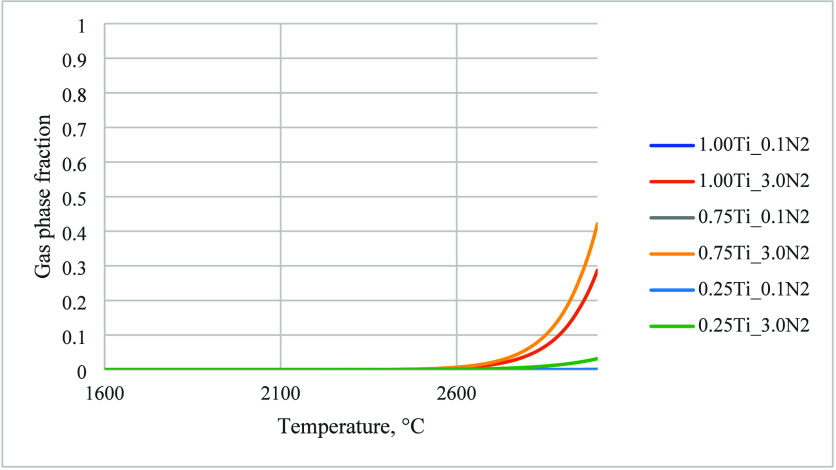
Evolution of gas phase molar fraction of Ti with temperature in nitrogen, with and without carbon, calculated using FactSage.

The discrepancy between FactSage calculations and experiments could be an indication of impurities interacting with each other and with carbon. The removal of impurities by heating, both its mechanisms and its kinetics, is an area that needs further research. This knowledge is especially relevant for electrically heated fluidized beds; the shorter residence time in fluidized beds, relative to fixed beds, means that the kinetics of impurity removal becomes more important.

### 4.3 Purity Analysis Techniques

Figure 3 and Table S1 (Supporting Information) show that significant differences exist with various analytical methods on key impurity concentrations in untreated graphite flakes. For example, when comparing GDMS results to those of ETV-ICP-OES, Al concentration was 50% lower and Si concentration was almost 10 times lower, while Mo and V concentrations were over 90 times higher. ICP-OES detected much higher amounts of Si, Fe, and Al. The results from PIXE were somewhat in between those of ICP-OES and GDMS.

Several factors may have contributed to these differences, such as sample inhomogeneity, analysis uncertainties, and measurement deviations. However, such significant differences, especially for major impurities such as Al and Si, are most likely due to the different technical approaches of these methods. It is worth noting that repeat analyses were done with the ICP-OES and PIXE methods, and repeatable results were obtained for both as shown in Supporting Information (Table S1).

Figure 3 shows that overall results from PIXE and solution-based ICP-OES are somewhat close to each other for both major and minor impurities. On the other hand, GDMS can detect more elements than PIXE and ICP-OES. PIXE does not detect Na, P, V, and Zr, while ICP-OES misses S, Ni, and Zn. The specific ETV-ICP-OES method used in this work is limited to 18 elements; however, ETV-ICP-OES can be used to detect other elements as well.

Table S1 and Figure 4 and Figure 5 show that significant disagreements also exist among different methods of the analysis of refined graphite.

Currently, many research publications rely on only one of the analysis methods, most commonly GDMS and ICP-OES. Since different graphite analysis methods may disagree on the exact concentrations of elements, as observed in this work, results using different analysis methods may not be comparable. There is thus a need to develop a common standard graphite purity analysis method that is reproducible across different laboratories.

GDMS is a method for analyzing the composition of solid samples and simultaneously performing a semiquantitative analysis of impurities.^36^ It should be noted that test sample inhomogeneity cannot be accounted for and may contribute to a substantial increase in the reported estimated uncertainties. One study showed that GDMS exhibited discrepancies by more than 1 order of magnitude for repetitive analyses of a series of trace components in the same sample.^37^ Furthermore, the results of different laboratories using the same kind of instrument are frequently not comparable. In our analysis, GDMS measures Ni at 94 ppm in the 2800M60 sample, nearly 16 times higher than the amount of Ni measured by GDMS (6 ppm) in the untreated natural graphite.

ICP-OES has the advantage that it can account for the inhomogeneity of the solid samples, though it is possible that the digestion process may not extract all of the elemental impurities because some graphite phases are resistant to wetting by a leaching chemical. Another advantage is that the standard solutions are relatively easy to prepare and can be measured very quickly. This leads to a further advantage of the ICP-OES method, which is that it gives consistent results if the samples are carefully prepared according to a standard protocol. This can be seen in the repeated analysis for the same samples in Table S1. A published study showed that the relative error of ICP with a sample amount of 1 g was ∼0.01%.^37^ However, ICP-OES requires that the samples be aerosolized, and due to the complicated sample preparation, the detection limit is typically higher for ICP-OES than for GDMS, PIXE, and ETV-ICP, though new approaches for sample preparation are being developed to bring the detection limit of ICP-OES close to that of other methods.^38^ In addition, many ICP-OES methods cannot detect sulfur, which is a major disadvantage. Finally, the accuracy of the final analysis results is restricted by sample preparation.

ETV-ICP-OES sidesteps many limitations of ICP-OES by eliminating the need for acid digestion and thus greatly simplifies sample preparation. This allows the ETV-ICP-OES method to rival GDMS in terms of the detection limit. As noted above, ASTM has developed a standard method for analyzing impurities in graphite using this method.

Among the four methods employed in this work, the PIXE method has the advantage of being the most cost-effective. Like ICP-OES, PIXE also exhibits very good repeatability, although it still shows inconsistencies. For example, it cannot detect Zr in untreated samples (Table S1) while it consistently detects this element in the purified ones. Saitoh et al. conducted a comparative study of PIXE, ICP-MS and ICP-OES with NIST standards.^39^ They found that the data repeatability of ICP methods was slightly better than that of PIXE, which our work has also shown to be the case. On the other hand, Saitoh et al. found that data from PIXE were more consistent with the NIST standards. As a result, Saitoh et al. recommended PIXE as the preferred analysis technique for environmental studies. PIXE has not been commonly used for graphite analysis. Among the various analysis methods used in this work, PIXE stands out for its low cost and good data repeatability. To the best of the authors’ knowledge, this is the first published work that used PIXE to determine impurity concentrations in high purity graphite.

Table 5 summarizes the advantages and disadvantages of the analysis techniques discussed above.

**Table 5:** Summary of the Four Graphite Analysis Techniques Used in This Work

analysis techniques	advantages	disadvantages
GDMS	Good detection limit for all elements, easy sample preparation, direct solid sample measurement	Higher uncertainties, very high cost, sample inhomogeneity may influence the results
ICP-OES	Better able to address sample inhomogeneity, good repeatability, speed	Cannot directly measure solid samples, complicated sample preparation techniques via acid leaching, poor detection limit
ETV-ICP-OES	Good detection limit for all elements, easy sample preparation, direct solid sample measurement	High cost
PIXE	Low cost, easy sample preparation, direct solid sample measurement, good repeatability	Detection limit varies

## 5 Conclusion

Canadian natural graphite flakes sized to three commercially viable particle size ranges were subjected to ultrahigh temperature heat treatment with a high temperature batch furnace operated by NRC and a continuous counterflow reactor operated by AETC. Four elemental impurities analysis techniques were used in this work. They were GDMS, PIXE, ICP-OES, and ETV-ICP-OES.

For the NRC reactor tests, the results show that natural graphite can be purified to >99.1 wt % carbon when heated to 2500 °C for 60 min or longer. The purity reaches >99.99 wt % carbon when heated to 2800 °C for 15 min or longer. Tests conducted with the NRC reactor show that better purification results are obtained with higher temperatures (2800 °C) held for shorter durations (15 min) than with lower temperatures (2500 °C) held for longer durations (120 min).

The purification tests conducted with the AETC reactor show that the natural graphite is purified to higher purity than that with the NRC reactor; the concentrations of nearly all impurities drop to below 15 ppm. A critical difference between the AETC reactor and the NRC reactor is that the graphite flakes are heated instantly to very high temperatures due to arcing generated in the AETC reactor, while in the NRC reactor, the flakes are heated gradually over hours in a stagnant crucible. Despite the residence time in the AETC reactor being similar to or shorter than that in the NRC reactor, the purification is much better. This confirms that faster heating and material fluidization are more effective for graphite purification possibly because carbide formation is inhibited. XRD analysis shows that nearly all samples, including the untreated ones, have good crystallinity, with an interlayer distance of ∼0.335 nm.

Regarding some of the recognized elements that are disruptive to Li-ion batteries, Mg, Al, and Cu are easily removed with heat treatment, even at a relatively moderate temperature of 2500 °C. To reduce Fe content to below 30 ppm, it is more effective to increase the temperature to 2800 °C. For Si removal, a higher temperature is also more beneficial.

The experimental results broadly agree with the FactSage calculations. FactSage calculations also confirm that to remove elements such as Fe and Si, a temperature of at least 2500 °C is needed with an adequate residence time. Further, some elements, such as Mo, cannot be removed by heat treatment according to FactSage, in agreement with our experimental observations.

This work provided further support for our choice for utilizing a fluidized bed for graphite purification because this approach leads to a much faster heating rate and much shorter residence time, thus vastly improving the efficiency of the high-temperature purification process.

## Supplementary Material



## Notes

Complete analytical results for both untreated and heat-treated graphite samples, obtained using four different analysis methods (GDMS, ICP-OES, ETV-ICP-OES, and PIXE); XRD scans of the Jumbo flake samples before and after the heat treatment (PDF)
