# Supplementary material for: Ultrahigh Temperature Purification of Graphite for the Development of a Continuous Process
Source: ACS Omega. 2025 Sep 16;10(38):44162–72. doi: 10.1021/acsomega.5c05566 (PMC12489646; doi:10.1021/acsomega.5c05566)
Supplement: Supplementary file 1 [file ao5c05566_si_001.pdf]

Supporting Information for the manuscript

Ultra-high temperature purification of graphite for the development of a continuous process

Yewen Tan[1*], Marc Duchesne[1], Anna Doninger[2], Matthew Meyers[2], Igor V. Barsukov[2]

[1]Natural Resources Canada, CanmetENERGY in Ottawa, 1 Haanel Drive, Ottawa, Ontario K1A 1M1, Canada

[2]American Energy Technologies Co., 265 Alice Street, Wheeling, IL 60090, USA

[*] Corresponding author, yewen.tan@nrcan-rncan.gc.ca

Note:

This document contains detailed analysis results of various samples, including untreated graphite and heat-treated graphite.

Table S1. Elemental analyses of the untreated graphite flakes reported in mg/kg. Duplicate or triplicate measurements were performed for ICP-OES and PIXE methods.

	GDMS			ETV-ICP-OES			ICP-OES			PIXE		
	**Untreated graphite flakes, medium**	**Untreated graphite flakes, large**	**Untreated graphite flakes, jumbo**	**Untreated graphite flakes, medium**	**Untreated graphite flakes, large**	**Untreated graphite flakes, jumbo**	**Untreated graphite flakes, medium**	**Untreated graphite flakes, large**	**Untreated graphite, jumbo**	**Untreated graphite flakes, medium**	**Untreated graphite flakes, large**	**Untreated graphite, jumbo**
Li	0.36	0.27	0.20	NA	NA	NA	NA	NA	NA	NA	NA	NA
Be	0.22	0.34	0.19	NA	NA	NA	NA	NA	NA	NA	NA	NA
B	1.10	1.70	1.10	NA	NA	NA	NA	NA	NA	NA	NA	NA
Na	4.60	1.50	2.60	NA	NA	NA	60/50	32/32	32/34/32	ND	ND	ND
Mg	290	250	210	NA	NA	NA	546/564	819/827	254/254/255	279/285	616/637	267/259
Al	2000	2000	1000	961	1164	567	4930/4890	5470/5380	2390/2530/2450	3100/3300	4460/4650	2430/2400
Si	8000	9000	5000	1041	851	476	13200/12500	9540/9580	4330/4580/4530	4870/5060	6140/6260	2800/2710
P	7.70	9.90	53	NA	NA	NA	38/36	41/34	45/31/31	ND	ND	ND
S	4000	4000	3000	2177	4324	4657	NA	NA	NA	1550/1530	1710/1600	2410/2510
Cl	0.59	ND	ND	NA	NA	NA	NA	NA	NA	NA	NA	NA

K	1.50	4.50	3.40	NA	NA	NA	579/572	589/587	198/197/198	333/313	436/412	214/226
Ca	7.60	16	29	25.8	23.7	18.8	333/328	956/841	434/504/399	215/237	396/387	279/280
Sc	0.03	0.03	0.01	NA	NA	NA	NA	NA	NA	ND	ND	ND
Ti	1.70	4.40	0.60	NA	NA	NA	80.5/74.8	127/127	41.8/38.8/40.2	60.9/57.1	62.9/62.0	83.9/78.8
V	1.70	1.60	0.67	165	81.0	39.3	43/45	48/49	20/19/20	40.9/41.5	22.9/22.9	ND
Mn	4.05	6.20	5.90	30.5	23.7	15.7	28.0/29.4	35.3/34.7	24.3/24.2/24.2	21.2/25.5	21.6/22.4	18.0/22.5
Fe	800	1000	900	918	858	886	4850/4600	4230/4100	4040/4160/4200	3320/3490	3070/3170	3220/3330
Co	0.13	0.10	0.20	1.80	1.80	2.00	ND	ND	ND	ND	ND	ND
Ni	6.10	9.00	6.40	5.10	7.60	9.60	ND	ND	ND	8.30/7.70	10.4/12.9	14.1/12.9
Cu	6.50	47.0	19.0	37.4	72.8	51.0	36.0/26.0	160/154	101/191/199	29.0/31.1	194/195	93.2/93.9
Zn	4.50	7.00	8.00	39.6	18.9	17.9	ND	ND	ND	9.20/10.6	23.1/22.5	15.5/14.6
Zr	0.11	ND	0.12	NA	NA	NA	13.7/13.9	20.9/19.7	18.9/18.3/18.8	ND	ND	ND
Mo	45.0	5.40	2.10	267	263	192	182/188	90.0/76.0	61.0/64.0/61.0	268/264	213/206	50.7/47.8

Note that ND denotes “not detected” where the element is below the detection limit, and NA denotes “not available” where the element was not analyzed.

Table S2. Elemental analysis of the jumbo flakes purified in the NRC furnace (concentration of impurities are listed in mg/kg)

Samples	Methods	Al	Si	Ti	V	Fe	Ni	Zr	Mo
**2800J15**	GDMS	3.00	2.00	0.48	0.08	0.07	ND	0.50	7.00
	PIXE	228	106	ND	ND	ND	ND	34.4	47.8
	ETV-ICP-OES	4.00	392	NA	6.50	49.9	20.1	NA	59.3
**2800J30**	GDMS	0.50	1.60	0.08	0.01	ND	ND	0.12	1.90
	PIXE	ND	ND	ND	ND	ND	ND	ND	ND
	ETV-ICP-OES	18.9	57.9	NA	6.10	38.3	18.3	NA	64.4
**2800J60**	GDMS	0.17	0.64	0.90	0.18	ND	ND	3.50	94.0
	PIXE	ND	ND	ND	ND	ND	ND	ND	ND
	ETV-ICP-OES	23.8	ND	NA	21.2	27.5	14.8	NA	65.7
**2500J30**	GDMS	0.17	310	1.60	1.30	210	2.20	0.24	2.80
	PIXE	ND	89.9	ND	ND	315	ND	ND	ND
	ETV-ICP-OES	11.4	27.4	NA	116	607	85.1	NA	104
**2500J60**	GDMS	0.64	1.00	0.46	0.20	0.05	ND	0.25	2.00
	PIXE	ND	124	29.4	ND	2.90	ND	24.7	52.6
	ETV-ICP-OES	16.4	ND	NA	42.0	58.0	35.1	NA	49.9

**2500J120**	GDMS	ND	1.20	0.45	0.16	ND	ND	0.15	3.5
	PIXE	ND	85.4	ND	ND	ND	ND	24.7	52.6

Table S3. Elemental analysis results of the medium flakes purified in the NRC furnace (concentration of impurities are listed in mg/kg)

Samples	Methods	Al	Si	Ti	V	Fe	Ni	Zr	Mo
**2800M15**	GDMS	0.56	1.70	ND	ND	ND	ND	ND	1.00
	PIXE	ND	90.6	ND	ND	1.80	ND	ND	62.3
	ICP-OES	ND	ND	ND	ND	ND	ND	ND	ND
**2800M30**	GDMS	1.10	1.10	0.08	0.02	ND	ND	ND	3.80
	PIXE	ND	ND	ND	ND	1.60	ND	ND	118
	ICP-OES	ND	ND	ND	ND	ND	ND	ND	ND
**2500M30**	GDMS	0.60	790	0.36	0.30	48.0	0.42	0.13	1.70
	PIXE	ND	1640	ND	10.2	520	ND	18.3	137
	ICP-OES	23.0	6370	59.3	31.0	2160	ND	6.90	204
**2500M60**	GDMS	0.79	940	2.30	1.50	76.0	0.68	0.24	18.0
	PIXE	ND	1730	21.7	11.7	485	2.10	14.4	127
	ICP-OES	14.0	4220	62.4	27.0	1130	ND	8.00	156
**2500M120**	GDMS	0.50	150	0.48	0.39	0.76	ND	0.13	3.10
	PIXE	ND	520	20.9	ND	15.9	ND	21.3	197
	ICP-OES	ND	ND	ND	ND	ND	ND	ND	ND

Table S4. Elemental analysis results for the samples purified in the AETC reactor (concentration of impurities are listed in mg/kg)

	GDMS			ETV-ICP-OES			PIXE		
Element	Medium	Large	Jumbo	Medium	Large	Jumbo	Medium	Large	Jumbo
Al	ND	ND	ND	20.2	ND	ND	ND	ND	ND
Si	0.72	0.48	2.10	3.40	8.60	3.40	ND	ND	ND
S	2.70	2.40	5.10	8.60	3.70	8.60	ND	ND	ND
Ti	ND	ND	ND	NA	NA	NA	ND	62.2	ND
V	ND	ND	ND	1.10	ND	0.08	ND	ND	ND
Fe	0.11	0.07	ND	3.20	8.00	13.9	ND	2.20	1.90
Ni	ND	ND	ND	0.36	ND	1.70	ND	ND	ND
Mo	ND	ND	ND	ND	0.20	0.20	ND	ND	ND
Mn	0.12	0.01	0.28	ND	ND	ND	5.80	0.00	0.00
Cr	ND	ND	ND	1.04	0.66	2.80	ND	ND	ND
Ca	ND	ND	ND	2.08	0.11	ND	ND	ND	ND

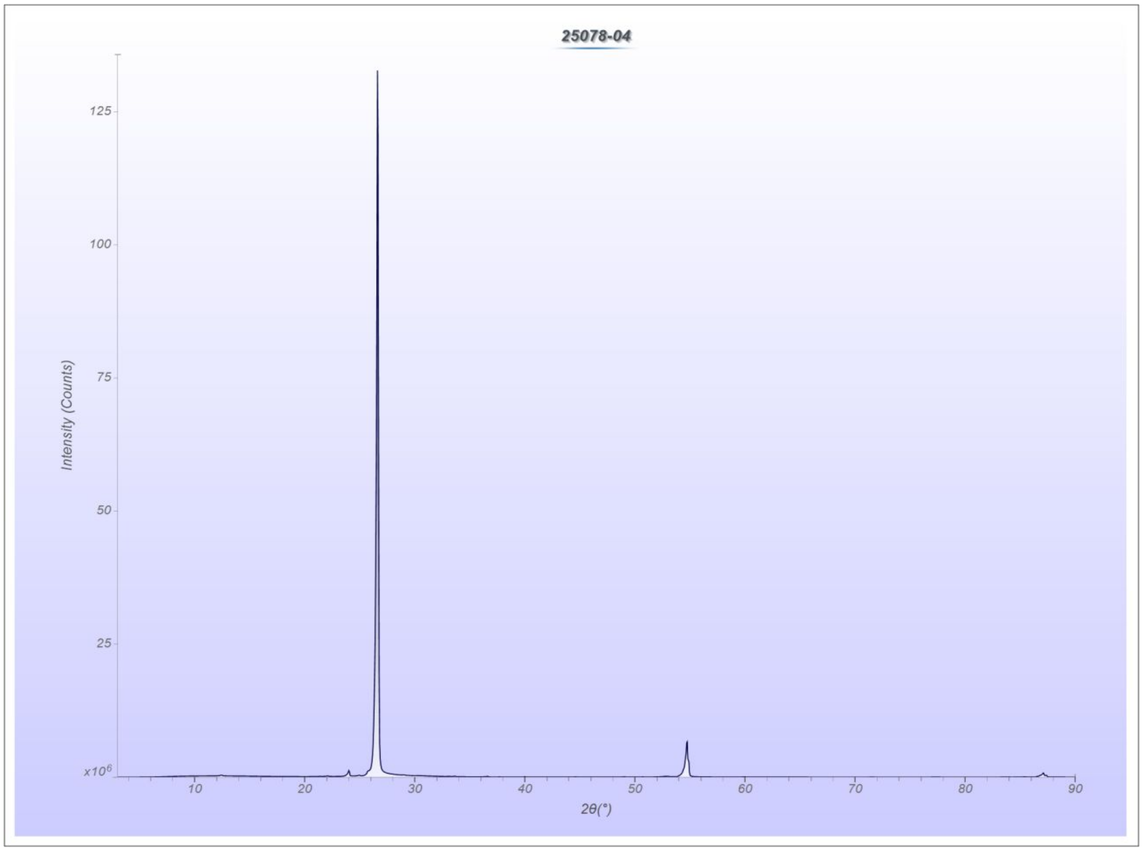

Figure S1. XRD scan results for the untreated jumbo flake graphite

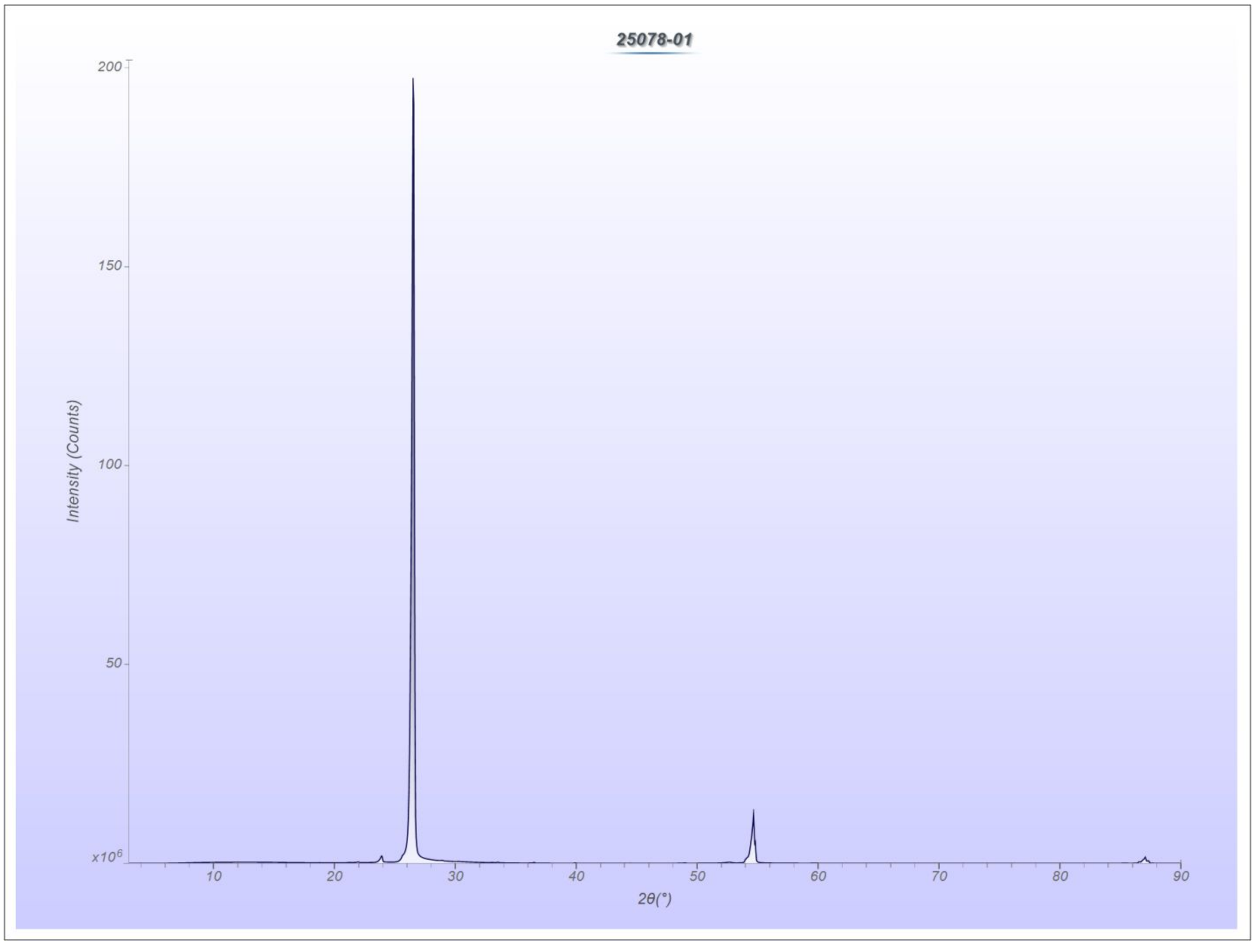

Figure S2. XRD scan results for the sample 2800J15

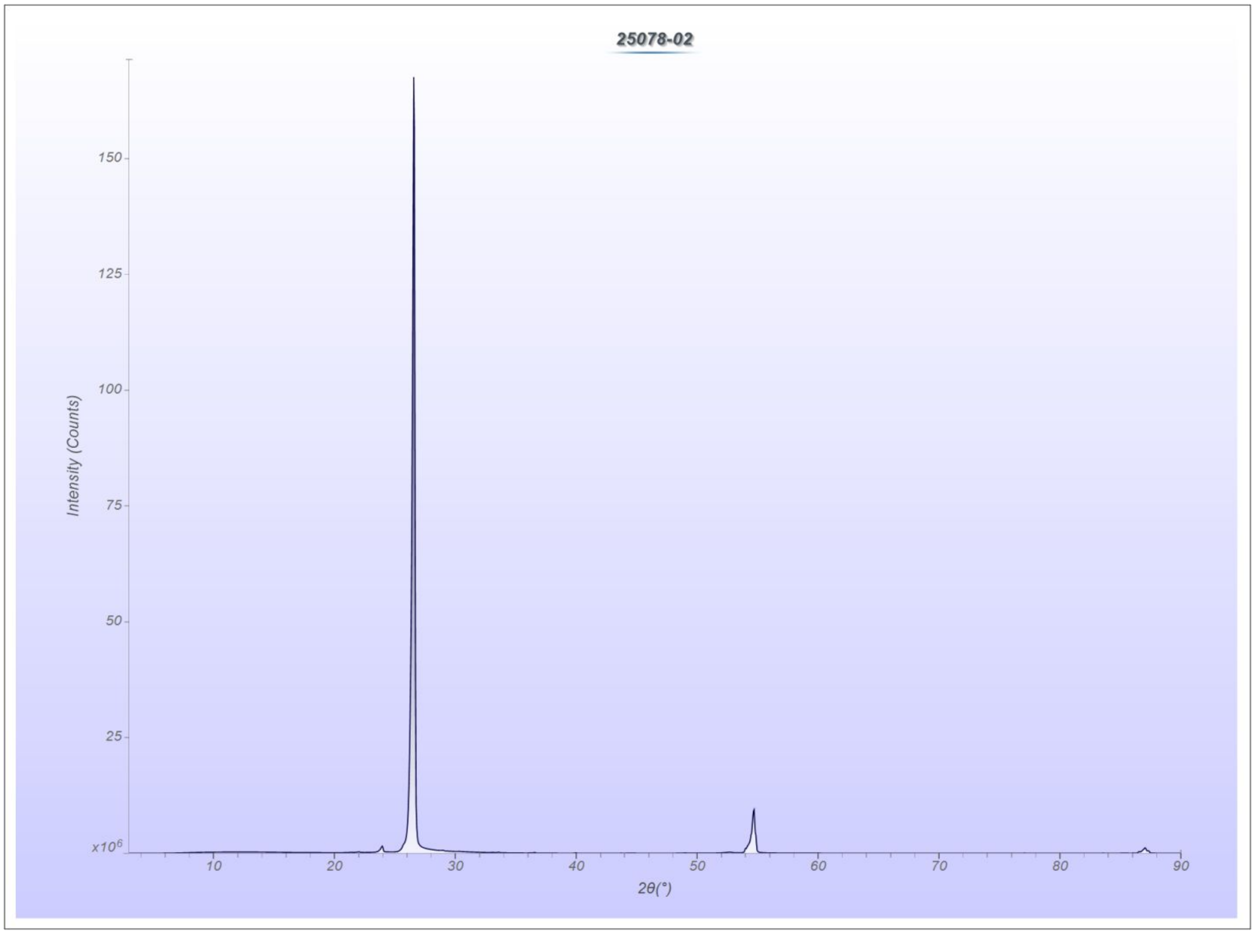

Figure S3. XRD scan results for the sample 2500J30

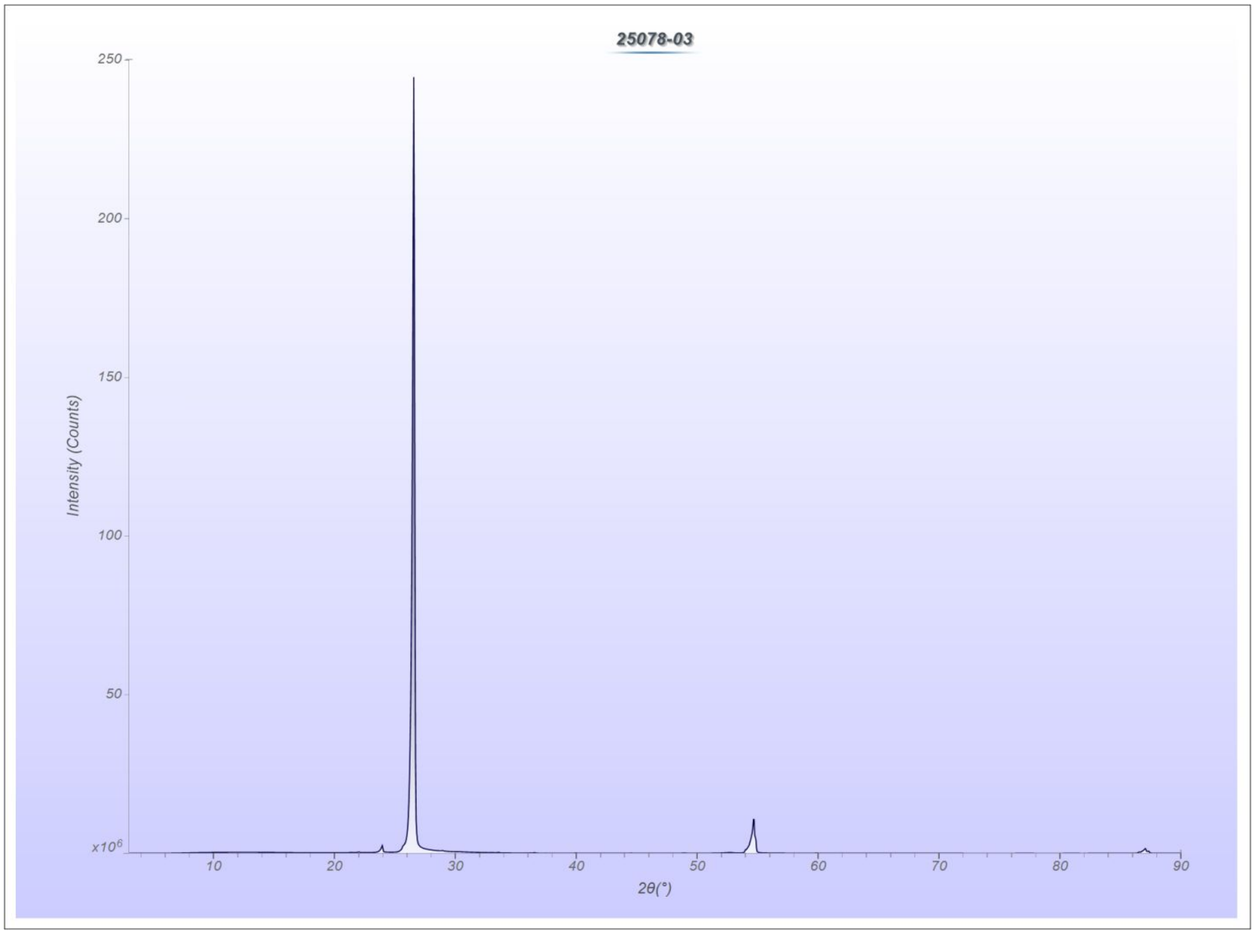

Figure S4. XRD scan results for the jumbo flakes treated in the AETC reactor
